# Supplementary material for: Continuous non-contact vital sign monitoring of neonates in intensive care units using RGB-D cameras
Source: Sci Rep. 2025 May 15;15:16863. doi: 10.1038/s41598-025-00539-9 (PMC12081704; doi:10.1038/s41598-025-00539-9)
Supplement: Supplementary file 1 — Supplementary Information. [file 41598_2025_539_MOESM1_ESM.pdf]

Continuous non-contact vital sign monitoring of neonates in intensive care units using RGB-D cameras

Silas Ruhrberg Estévez[1,*], Alex Grafton[1], Lynn Thomson[2], Joana Warnecke[1], Kathryn Beardsall[2,3], and Joan Lasenby[1]

[1]Department of Engineering, University of Cambridge, Cambridge, UK
[2]Rosie Hospital, Cambridge University Hospitals NHS Foundation Trust, Cambridge, UK
[3]Department of Paediatrics, University of Cambridge, Cambridge, UK
[*]Corresponding author: Silas Ruhrberg Estévez, sr933@cam.ac.uk

1 Flow-volume loops

A representative example of a single flow volume loops calculated from both camera and ventilator data is shown in Figure 1. The two loops show similar characteristics. The camera system is sensitive enough to capture subtle variations in breathing such as interrupted breaths. Interruptions occur when a neonate attempts to breathe during a ventilator-induced breath, which is then terminated prematurely. An example of a prematurely terminated breath in the ventilator data and a terminated breath followed by a second autonomous breath in the camera data is shown in Figure 2.

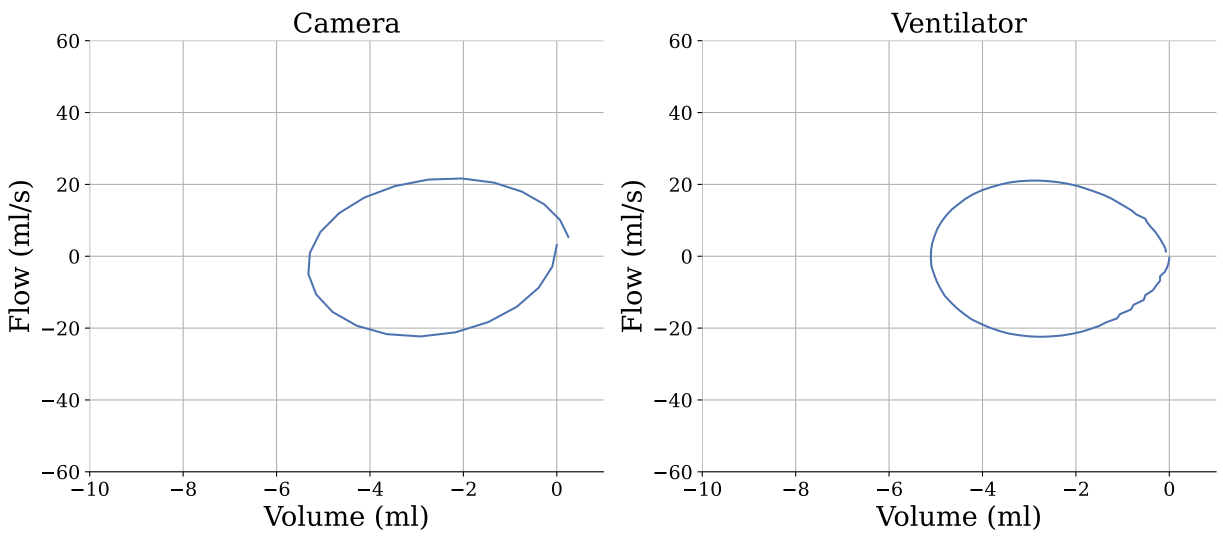

Supplementary Figure 1. Representative single loop from matched breaths in ventilator and camera data.

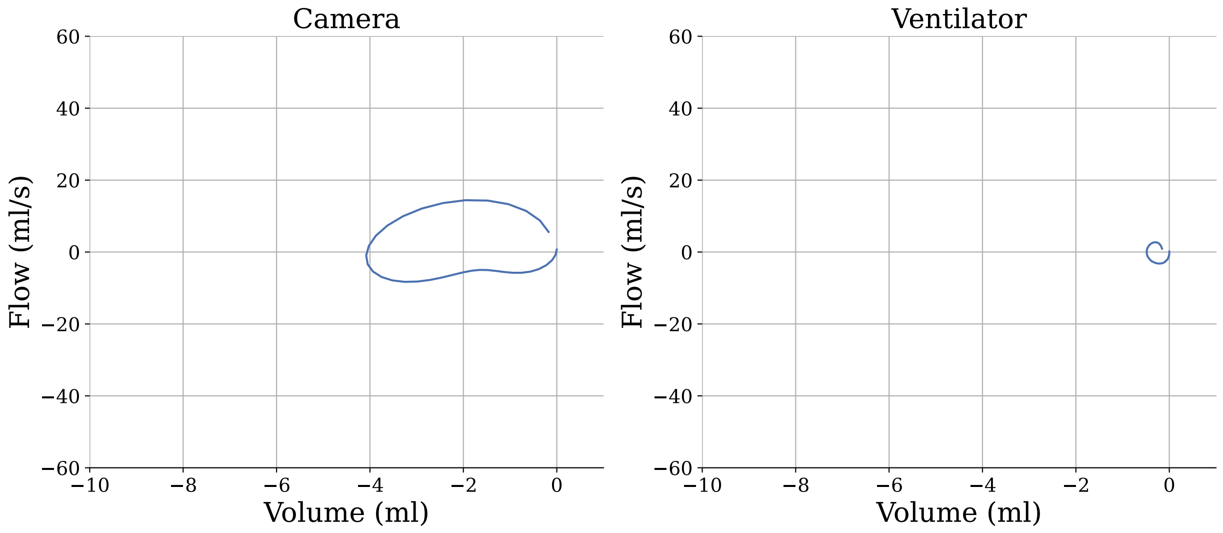

Supplementary Figure 2. Single flow-volume loop generated using camera derived respiratory signal and flow data from the ventilator reveals a prematurely terminated ventilator breath followed by an autonomous breathing attempt.

2 Breathing asymmetry

The spatial distribution of the breathing efforts can be monitored using the camera approach. For this, two of the segments of the region of interest, corresponding approximately to the left and right thorax regions are considered. Respiratory signals are computed for each region, and the effective tidal volumes of these signals are compared to identify breathing asymmetries. Asymmetry in breathing was defined as

$$A = \frac{L - R}{(L + R)/2} \cdot 100\%, \tag{1}$$

where L and R are the effective tidal volumes of the respiratory signal derived from the left and right sides of the thorax, respectively. This asymmetry metric A provides a quantitative measure of the imbalance in breathing efforts between the left and right sides of the thorax.

However, no neonates with known congenital scoliosis or pneumothorax were part of this study. To demonstrate the sensitivity of the system, we instead compared the resulting signals which arise when part of the chest region is covered by medical equipment which mimics the lack of motion observed during a pneumothorax. As expected, the asymmetry score is much higher in a baby with parts of the chest covered compared to a baby where the entire chest area is visible (see Fig. 3).

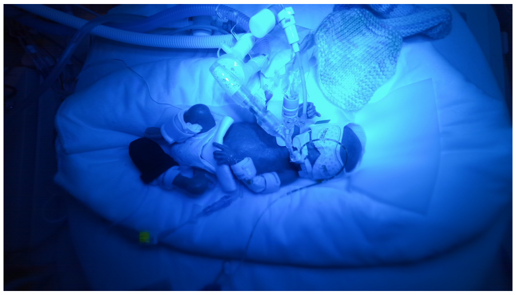

(a) Equipment covering chest area

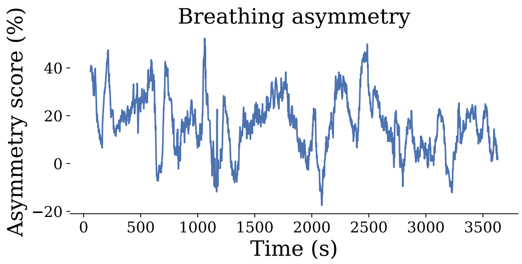

(b) Large asymmetry

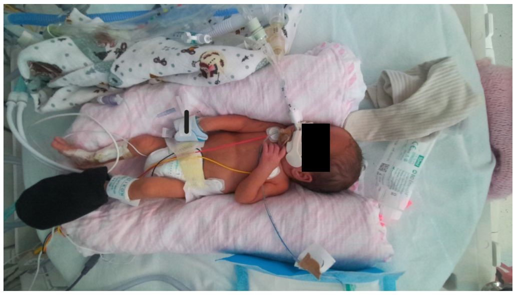

(c) Chest area uncovered

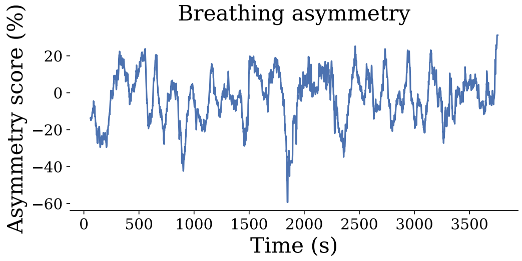

(d). Small asymmetry

Supplementary Figure 3. Proof of concept of asymmetry monitoring. A neonate recorded with parts of the chest covered by medical equipment (a) results in a strong asymmetry (b) whereas a mainly uncovered chest (c) results in symmetric breathing (d). The temporal variations in the asymmetry signal are due to hand movements in front of the chest.

3 ECG recording editing

**Adjusted Time (s)**	**0**	**2**	**4**	**6**	**8**	**10**	**12**
**ECG rate example 1 (bpm)**	158	160	161	**211**	164	165	166
**ECG rate example 2 (bpm)**	147	147	145	**126**	**126**	141	141

Supplementary Table 1. Representative examples of non-physiological changes in heart rate measured by the ECG that were manually excluded from the data (bold).

4 Haemoglobin absorption

Peripheral oxygen saturation (SpO_2) is defined as the proportion of haemoglobin in the blood that is bound to oxygen [1]. It is calculated as

$$SpO_2 = \frac{[HbO_2]}{[HbO_2] + [Hb]} \cdot 100\%, \tag{2}$$

where $[Hb]$ and $[HbO_2]$ represent the concentrations of non-oxygenated and oxygenated haemoglobin in the blood, respectively.

Non-invasive methods for monitoring oxygen saturation measure the different light absorbance properties of haemoglobin depending on its oxygenation state (see Fig. 4). Traditional pulse oximeters use dual-wavelength LEDs in the red and infrared range due to significant differences in absorption between oxygenated and deoxygenated haemoglobin. This principle has been extended to non-invasive imaging systems utilising

dual-wavelength cameras [2]. There have also been approaches using RGB cameras that exploit differences in light absorbance between blue and red wavelengths [3]. The camera used in this study is a versatile platform for oxygen saturation detection, as it can utilise both RGB and infrared imaging methods.

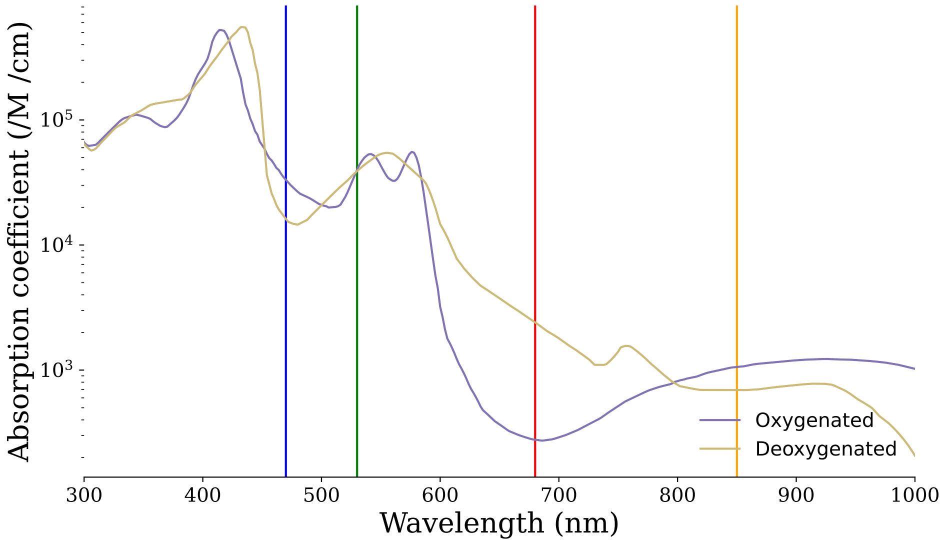

Supplementary Figure 4. Absorption spectra of oxygenated and non-oxygenated haemoglobin. Figure produced using data from [4]. Approximate locations of red, green, blue and infrared wavelengths are indicated with vertical lines. Absorption differences between oxygenated and deoxygenated haemoglobin are observed for blue, red and infrared wavelengths.

5 Pulse oximeter waveform classification

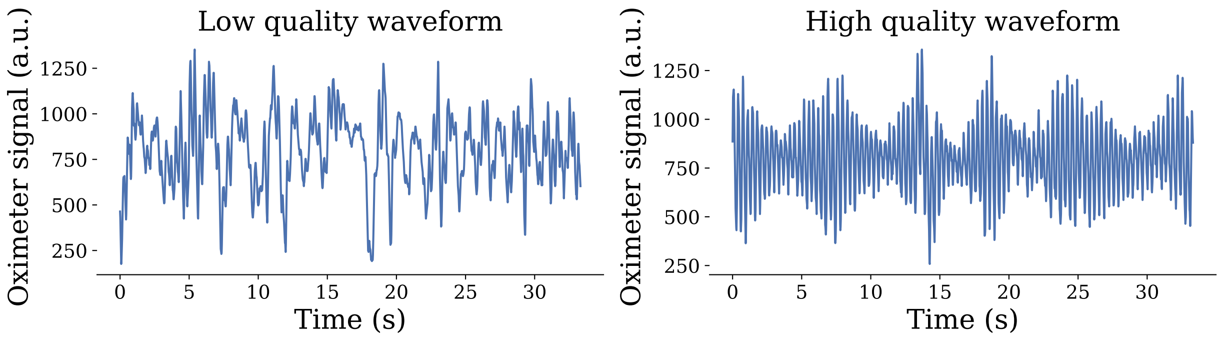

Supplementary Figure 5. Representative examples of high- and low-quality pulse oximeter waveforms. The high-quality waveform exhibits clear periodicity, characterised by regular oscillations. In contrast, the low-quality waveform is irregular, containing spikes and lacking distinct oscillatory patterns. The signals represent a combination of colour signals and are displayed in arbitrary units (a.u.).

6 Oxygen saturation annotations

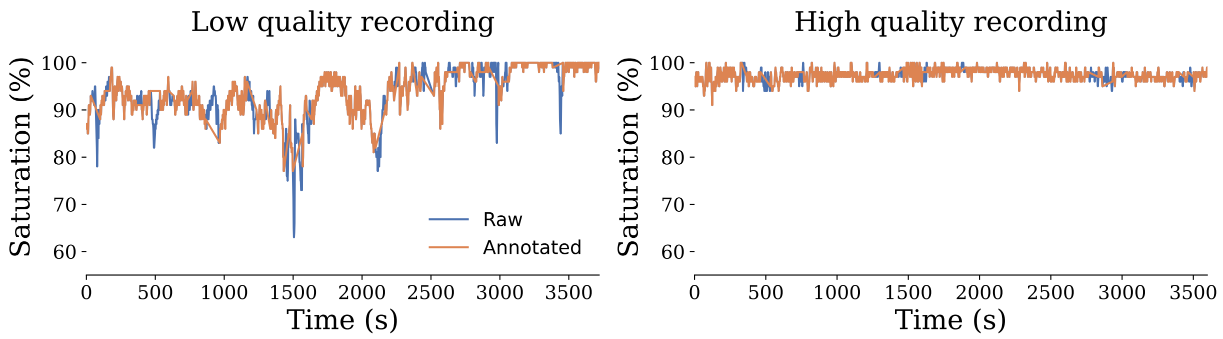

Supplementary Figure 6. Annotated oxygen saturation recordings from two representative neonates. High-quality recordings, characterised by stable and consistent waveforms, require minimal exclusion. In contrast, low-quality recordings, often attributed to neonatal movements, necessitate extensive annotations.

References

1. Kim, Y., Ganduglia-Cazaban, C., Chan, W., Lee, M. & Goodman, D. C. Trends in neonatal intensive care unit admissions by race/ethnicity in the United States, 2008–2018. *Scientific Reports* **11,** 23795 (Dec. 2021).

2. Shao, D. *et al.* Noncontact Monitoring of Blood Oxygen Saturation Using Camera and Dual-Wavelength Imaging System. *IEEE Transactions on Biomedical Engineering* **63,** 1091–1098 (2016).

3. Bal, U. Non-contact estimation of heart rate and oxygen saturation using ambient light. *Biomed Opt Express* **6 (1),** 86–97 (Jan. 2015).

4. Prahl, S. *Optical absorption of hemoglobin* `http://omlc.ogi.edu/spectra/hemoglobin/index.html`. (accessed May 16, 2024).
